# Post-tracheostomy With Long-Standing Temporomandibular Joint (TMJ) Ankylosis Using a Cone-Beam Computed Tomography (CBCT) Scan for Airway Assessment: A Case Report

**DOI:** 10.7759/cureus.101662

**Published:** 2026-01-16

**Authors:** Vamsi Krishna Uppalapati, Rama Shankar, Deb Sanjay Nag, Prashant Sharma, Roshan Gope, Vissapragada Rama Murthy

**Affiliations:** 1 Anesthesiology, Tata Main Hospital, Jamshedpur, IND; 2 Oral and Maxillofacial Surgery, Tata Main Hospital, Jamshedpur, IND; 3 Anesthesia and Critical Care, Tata Main Hospital, Jamshedpur, IND

**Keywords:** awake foi, cbct, difficult airway, tmj ankylosis, tracheal stenosis

## Abstract

Cone-beam computed tomography (CBCT) scans offer a static picture of airway anatomy and help diagnose anatomical or pathological anomalies. Patients with post-tracheostomy status have a high probability of tracheal stenosis. The airway must be thoroughly examined before induction. Three-dimensional visualization using CBCT allows the anesthesiologist to make informed decisions regarding the intubation approach and appropriate endotracheal (ET) tube size, thereby reducing the risk of perioperative complications.

In this case report, we discuss the role of 3D imaging in assessing the probability of tracheal stenosis in a 56-year-old patient with a 40-year history of long-standing temporomandibular joint (TMJ) ankylosis.

## Introduction

Tracheal stenosis can be caused by factors such as airway trauma, post-surgical airway changes, airway tumors, and congenital factors [1], making it difficult to negotiate an adequately sized endotracheal (ET) tube [2]. It is of utmost importance to rule out tracheal stenosis before the induction of anesthesia to avoid perioperative mortality and morbidity in such complex scenarios.

Subglottic stenosis is a life-threatening emergency complication in patients with post-tracheostomy status. Temporomandibular joint (TMJ) ankylosis, in combination with post-tracheostomy status in a patient scheduled for elective laparoscopic surgery under general anesthesia, represents an anticipated difficult airway based on a prior history of traumatic intubation and therefore requires proper workup and meticulous planning for airway management [3].

We present a patient with TMJ ankylosis secondary to trauma for 40 years, presenting with restricted mouth opening, limited neck extension, and a history of tracheostomy with subsequent decannulation. It was imperative to assess for the presence of tracheal stenosis during the pre-anesthesia check-up to avoid multiple attempts at securing the airway. After extensive discussion and review, a decision was made to recommend a cone-beam computed tomography (CBCT) scan, with the aim of visualizing potential underlying subglottic stenosis [4].

CBCT utilizes a low-power medical fluoroscopy tube that enables continuous imaging during the entire scan. Traditional CT uses a fan-shaped X-ray beam to capture data onto image detectors arranged in an arc around the patient, generating a single-slice image for each scan. To accurately reconstruct the images, each slice must overlap slightly. In contrast, CBCT technology employs a cone-shaped X-ray beam that transmits onto a solid-state area sensor for image capture, generating a whole-volume image in a single rotation. CBCT is faster and can be performed at a lower radiation dose due to the lack of overlap between slices; additionally, three-dimensional imaging provides airway measurements that help the anesthesiologist determine the appropriate ET tube size and intubation approach [5].

## Case presentation

**Figure 1:**
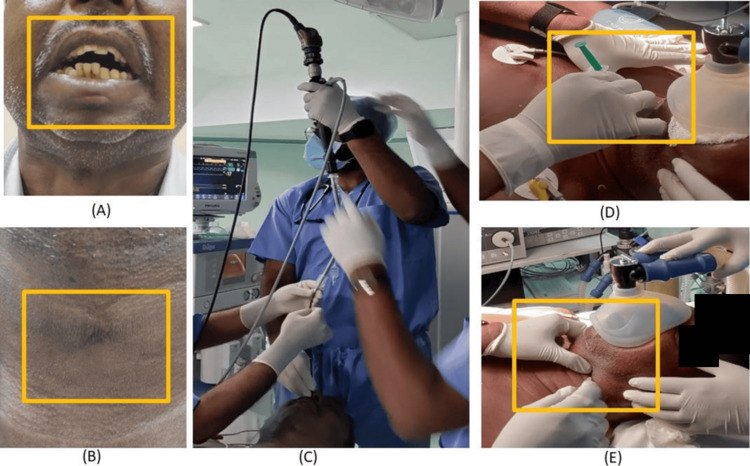
(A) Restricted mouth opening; (B) post-tracheostomy status; (C) fiberoptic intubation (FOI) intubation process; (D) and (E) airway blocks

A 56-year-old male with chronic cholecystitis was scheduled for an elective laparoscopic cholecystectomy. The patient had a history of trauma causing panfacial fractures approximately 40 years ago, which led to TMJ ankylosis over time. Figure 1 illustrates the severity of TMJ ankylosis.

Due to TMJ ankylosis, the patient had limitations in dietary habits, resulting in chronic malnutrition. A corrective TMJ ankylosis surgery was attempted about eight years ago. The patient landed in a Cannot Ventilate, Cannot Intubate (CVCI) scenario [6], which necessitated an emergency tracheostomy, as shown in Figure 1, performed as a life-saving procedure, and the subsequent surgery differed.

**Figure 2:**
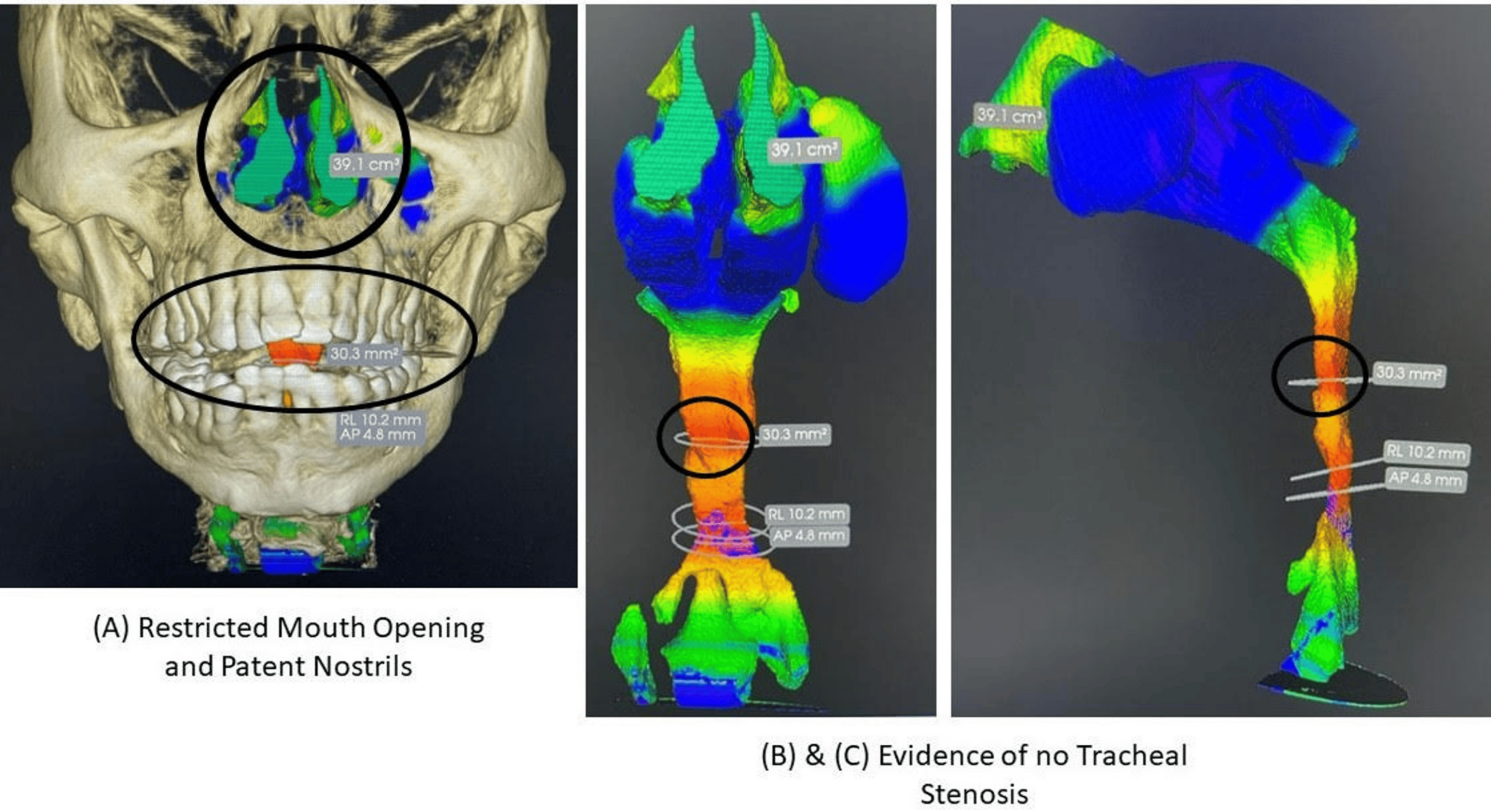
CBCT-3D imaging of airway with measurements (A) Sagittal view showing the anteroposterior diameter of the trachea at the subglottic level; the narrowest point is in the transverse tracheal diameter. (B) Axial view illustrating the bone wall in the oblique plane. (C) Axial mode with a three-dimensional reconstructed image, showing the morphology of the airways. Measurement markers indicate the locations where dimensional assessments were made. CBCT, Cone-beam computed tomography

With the above complex history, the patient arrived for an elective laparoscopic cholecystectomy at Tata Main Hospital, Jamshedpur, India. The patient was evaluated using a multimodal approach, concluding that an anticipated difficult airway scenario was present. Thus, the patient was referred for CBCT scanning for better visualization of the tracheal anatomy. A CBCT image was acquired following a standardized protocol, with the patient in an upright position. Multiplanar reconstruction was applied in the axial, sagittal, and coronal planes to obtain airway measurements. The lowest anteroposterior and transverse diameters of the trachea were measured at preset anatomical sites, such as the subglottic area and mid-trachea. Measurements were taken in millimeters using the in-built measurement tools of the imaging software. To ensure consistency, the measurements were reviewed by two clinicians, and the average values were entered into the analysis. CBCT images that reveal the tracheal airway were displayed in a 3D view (Figure 2).

With evidence from CBCT 3D scan images, we concluded that the patient’s tracheal dimensions were within the normal range, with no stenosis. However, the patient presented with limited mouth opening, prompting us to plan awake fiberoptic intubation (FOI), in accordance with ASA-2020 airway guidelines [7]. The patient signed a written informed consent form, through which they gave permission for this case report and related images to be published.

The airway management plan was developed based on the CBCT results, which showed a tracheal size in the normal range and no significant stenosis, allowing the use of awake FOI under conditions that would not result in unexpected airway deterioration.

## Discussion

About eight years ago, our patient underwent an emergency tracheostomy, likely resulting in tracheal stenosis. The patient also presented with restricted mouth opening due to severe TMJ ankylosis. Hence, awake FOI intubation is recommended, per ASA-2020 airway guidelines, during the brainstorming session about securing the airway in the possibility of tracheal stenosis, to avoid multiple attempts with various-sized ET tubes. Extensive literary research on tracheal radiological anatomy and measurements was carried out. As Kochhar et al. (2021) [8] presented in their article, a CBCT scan is one of the suggested solutions for tracheal measurement, along with a 3D view of nostrils and mouth opening measurements. Standard transverse internal tracheal diameter is between 15 and 25 mm in men and 10 to 21 mm in females, with a cross-sectional area between 250 and 350 mm² and a volume between 30 and 40 cm³ [9]. Hence, the CBCT scanning was carried out, and the patient’s measurements were satisfactory for awake FOI [10] and ET intubation, as shown in Figure 2.

The use of CBCT in this study was due to its capability of creating high-resolution, 3D images of airway structures, using comparatively low doses of radiation and short scan times, despite the fact that conventional CT is deemed the standard imaging modality for assessing airway structures in detail. CBCT has found more applications in maxillofacial and airway examination, especially where accurate dimensional measurement is needed. There are, however, weaknesses of CBCT, such as diminished soft-tissue contrast compared with standard CT, as well as uncertain variability in the accuracy of measurements with changes in acquisition parameters. These restrictive factors were taken into account throughout the process of interpretation; the results of CBCT were compared with the clinical evaluation to manage the airways.

The patient and his family members were briefed about the anticipated perioperative risks in the possibility of tracheal stenosis. The patient and his family had undergone a traumatic experience eight years ago during TMJ ankylosis correction. High-risk-informed written consent was obtained accordingly. The operating theater was arranged with a difficult airway cart, consisting of all-sized flexometallic ET tubes, an emergency cricothyrotomy set, and a well-functioning suction apparatus. Before shifting to the operating theater, the patient was nebulized with xylocaine (2%), and standard ASA monitors were connected once the patient was wheeled in. Injection midazolam (1 mg) and fentanyl (50 mcg) were given as premedication. Injection glycopyrrolate (0.2 mg) was given as an antisialagogue.

Our CBCT scans show a low probability of tracheal stenosis. Hence, we attempted awake nasal FOI with a 7.0-size flexometallic ET tube. For better patient tolerance and cooperation, airway blocks were administered. The superior laryngeal nerve block was performed via an external approach to identify the hyoid bone. The needle was walked inferiorly along the hyoid bone, injecting 2% lidocaine (2 mL) on either side, thereby blocking the internal and external branches. The recurrent laryngeal nerve block was performed at the level of the thyroid cartilage using a 22-gauge needle, penetrating the cricothyroid membrane with 4% lidocaine (5 mL).

With the help of CBCT scans and tracheal measurements, a 7.0-size ET tube was the correct choice. As the FOI scope visualized the vocal cords and tracheal rings, the adequately sized tube, which had been railroaded prior, was advanced into the trachea. After confirmation of the ETCO₂ trace and auscultation, the patient was induced with propofol (150 mg) and fentanyl (150 mg) and paralyzed with vecuronium (8 mg). To prevent postoperative nausea and vomiting, injections of dexamethasone (8 mg) and ondansetron (4 mg) were given. The procedure lasted one hour and was uneventful. The patient was adequately reversed with neostigmine (3,300 mcg) and glycopyrrolate (650 mcg) and shifted to recovery with a T-piece after thorough monitoring. The patient was extubated in a controlled environment after one hour.

The study has a limitation, being narrow in the scope of the patients, and the results cannot be extended to all patients with expected difficult airways. Moreover, due to the ability to visualize airway anatomy in detail through the use of CBCT, its role should be viewed as an addition to existing imaging modalities and clinical airway examination, but not as an alternative. The research will require additional investigations to enhance standardized guidelines and clinical pointers for the use of CBCT in airway assessment.

Although some existing studies have considered airway evaluation with standard imaging tools, the literature describing the utilization of CBCT in the evaluation of tracheal dimensions in patients with predicted challenging airways is scarce. The case indicates that CBCT can be used as an adjunctive imaging method to assess the airway preoperatively, when it is necessary to evaluate complex airway anatomy or previous traumatic airway experiences. The current report adds practical value to the topic of individualized airway management devices by demonstrating how CBCT findings contributed to airway planning for the patient under consideration.

## Conclusions

This case highlights the potential utility of CBCT as an adjunctive tool for airway assessment in selected patients. CBCT is a revolutionary airway evaluation tool. While traditionally associated with dental imaging, its robust capabilities are pertinent to anesthesia. The field of anesthesiology benefits immensely by adopting CBCT, especially when confronted with challenging intubation scenarios such as restricted mouth opening, tracheal stenosis, and other predicted difficult airway situations. For anesthesiologists, CBCT offers an enhanced understanding of complex airway structures and provides a potential game-changer in pre-operative planning, promising to reduce complications and multiple intubation attempts significantly. As a research and teaching tool, CBCT promises a new era of precision and safety in anesthesiology.
